# Does peer review have a future?

**DOI:** 10.1093/jhps/hnz015

**Published:** 2019-05-06

**Authors:** Richard (Ricky) Villar

**Affiliations:** Editor-in-Chief, Journal of Hip Preservation Surgery

There appears to be a growing trend in the academic arena that is making me increasingly unsettled. The trend is for editors to reject a submission without peer review. That is something I have only once done, after more than a decade of editing, and even then, the process troubled me. I have returned a few papers to their authors because the English made no sense but have always encouraged resubmission once the language is better. But this trend is perhaps a sign of increasing journal desperation. As the number of peer-reviewed journals has increased by 3.5% year-on-year for the past 200 years [1], so editors have found it harder to find sufficient, decent reviewers.

For *JHPS* we are blessed and fortunate, as the quality and willingness of our reviewers has remained undiminished since our very first issue. Thank you to the so many who are involved. Yet, other journals may not be so fortunate so one can perhaps understand why journals seek to lighten the reviewer load, for fear of losing reviewers completely.

Look at the figures. In 2015, Kovanis *et al*. [2] reported that across a range of journals, the supply of submitted papers exceeded reviewer availability by between 15 and 249%. Furthermore, 20% of the researchers undertook 69–94% of the reviews. Perhaps more shocking was their finding that each year 63.4 million hours were devoted to peer review, of which 18.9 million hours were undertaken by the top 5% of contributing reviewers. It appears that the reward for providing a thorough review is to receive more requests rather than thanks. I well recall, years back when I was acting for another journal, receiving 128 papers for review in a single year, and that was not the journal’s record.

To me, if a journal wishes to practise immediate rejection, then why not practise immediate acceptance, too? Indeed, is it time to consider whether a paper needs review on submission in the first place?

Now I happen not to believe that at all, but you can see the drift, and why peer review is evolving in response to these challenges [3]. There is cascading review, where a rejected manuscript is transferred to another journal, along with the manuscript’s original review. And there is post-publication peer review (PPPR), an area that is expanding furiously and is having a dramatic impact on science overall. It is quicker than traditional peer review and has been made simpler thanks to the Internet and to various platforms dedicated solely to PPPR [4]. The principle of PPPR is that all research deserves the chance to be published, subject to some initial editorial selection process, and is an example of decoupling peer review from publishing [5].

The use of artificial intelligence is also developing in scientific publishing and is already being used to help identify new peer reviewers, identify bad reporting, detect plagiarism, bad statistics and fabricated data. The scientific community is not far off fully automated paper reviews [6]. Somehow, I feel that is a shame.

Anyway, be reassured, that all papers submitted to *JHPS* will be reviewed unless there is some totally unexpected circumstance. Sadly, we are unable to publish everything. It stands to reason that we are continually looking at the peer review process, and whether it can be changed and improved, not only for the benefit of our authors, but for our reviewers, too. Reviewers are, after all, the unsung heroes and heroines of so many scientific journals, and especially of *JHPS*.

Turning to look in detail at our last issue of *JHPS*, issue 5.4, again I enjoyed each article and it is always hard for me to separate out the few. Yet how about the excellent piece by Atzmon *et al*. [7] on the graft choices for acetabular reconstruction? Utterly brilliant, and immensely helpful, in my view. I had not realized there were so many options available until I read their paper—ligamentum teres (I will reserve personal judgement on that as a choice), iliotibial band, gracilis, quadriceps, semitendinosus, tensor fascia lata and plenty more.

I enjoyed also the study by Sutton *et al*. [8] on so-called ptosis of the hip, a newly described phenomenon seen on the AP radiograph of the hip that is essentially a reverse break in Shenton’s line. In this era of hi-tech investigation, there is so much information an experienced practitioner can gain from a plain radiograph. Simple investigations should never be forgotten when it comes to a thorough patient assessment.

And for this issue, issue 6.1, I am again spoiled for choice. However, and in part because of our increased involvement with open hip surgery, I was fascinated to read the paper from Denmark by Boje *et al*. [9], who looked at changes in pain to see if they might be associated with changes in quality of life and hip function two years after periacetabular osteotomy. They found that 84% of their patients were satisfied with the result of their surgery two years afterwards and would have gone through the operation again had they known the result in advance. That is a good result by any standards, I would suggest.

I was also interested to read the paper by Kim *et al*. [10], on the prevalence of radiologic acetabular dysplasia in asymptomatic Asian volunteers. They estimated a high prevalence of acetabular dysplasia in pain-free Asian hips. That is a finding well worth keeping in mind in our truly global era. Anyone from any nation can walk into a consulting room to seek our advice these days, wherever we may be practising.

So, as ever, please enjoy this issue of *JHPS*. It is published for you, the hip preservation practitioner, and is filled from cover to cover with brilliance. I commend this issue to you in its entirety.

My very best wishes to you all.
